# Ergänzender Einsatz von breitbandigen Absorbanzmessungen zur Klassifizierung einer Normalhörigkeit

**DOI:** 10.1007/s00106-025-01704-2

**Published:** 2025-12-22

**Authors:** Milena Walter, Marie-Christin Quandt, Alexander Mewes, Kevyn Kogel, Matthias Hey

**Affiliations:** 1 Medizinische Fakultät, Christian-Albrechts-Universität zu Kiel, Kiel, Deutschland; 2 Klinik für Hals-Nasen-Ohren-Heilkunde, Kopf- und Halschirurgie, Audiologie, Universitätsklinikums Schleswig-Holstein (UKSH), Campus Kiel, Arnold-Heller-Str. 3, Haus B1, Kiel, Deutschland; 3 MED-EL Elektromedizinische Geräte Deutschland GmbH, Starnberg, Deutschland

**Keywords:** Mittelohr, Sprachaudiometrie, Akustische Impedanzuntersuchungen, Reintonaudiometrie, Hörtests, Middle ear, Speech audiometry, Acoustic impedance tests, Pure tone audiometry, Hearing tests

## Abstract

**Hintergrund:**

Die Definition der Normalhörigkeit nach DIN-Norm bezieht sich auf die Luftleitungsschwelle der Tonaudiometrie. Die Mittelohrfunktion wird hierbei jedoch nicht gesondert berücksichtigt. Ziel der vorliegenden Studie war die prospektive Erhebung audiometrischer Kenndaten für Normalhörigkeit, welche nicht nur auf der Tonaudiometrie basiert, sondern mit objektiven Daten zur Mittelohrfunktion ergänzt wurden.

**Methode:**

Die Untersuchungen wurden an 84 Ohren tonaudiometrisch normalhörender Proband*innen im Alter zwischen 18 und 25 Jahren durchgeführt. Die objektive Mittelohrdiagnostik erfolgte unter Verwendung der klassischen Tympanometrie, der Breitband-Absorbanz-Tympanometrie und der Stapediusreflexschwellenmessung. Ergänzend wurde eine Messung der distorsiv produzierten otoakustischen Emissionen (DPOAE) durchgeführt.

**Ergebnisse:**

Bei 7 der 84 untersuchten Ohren fand sich eine Compliance von mehr als 2 mmho. Ergänzend zu den normalhörenden Ohren wurden diese im Weiteren separat betrachtet. Die Resonanzfrequenz dieser sieben Ohren lag unterhalb des ersten Quartils der Vergleichsgruppe (759 Hz), und die Absorbanz dieser Gruppe befand sich für Frequenzen bis 750 über dem 3. Quartil der Absorbanz für die Gruppe mit regulärer Compliance. In der Gruppe mit erhöhter Compliance waren in mehr Fällen als in der Vergleichsgruppe (14 % vs. 5 %)bei mind. einer Frequenz keine DPOAE nachweisbar. Bei 6 der 7 Ohren mit erhöhter Compliance fanden sich in allen gemessenen Frequenzen Stapediusreflexschwellen im Bereich der Whisker der Gruppe mit regulärer Compliance.

**Schlussfolgerung:**

In der DIN-Norm zur Klassifizierung von Normalhörigkeit besteht die Definition der Normalhörigkeit allein aufgrund tonaudiometrischer Parametrisierung. Die in dieser Studie gemessenen Referenzwerte in Bezug auf die Compliance des Trommelfells zeigen eine deutliche interindividuelle Varianz. Die Ergänzung zur Einschätzung der Mittelohrfunktion, wie die Messung der Compliance, der Resonanzfrequenz und/oder der Absorbanz-Verläufe, könnte zusätzliche Informationen zur Absicherung einer Normalhörigkeit liefern.

## Abstract (en)

**Background:**

The definition of normal hearing according to the DIN standard refers to the air conduction threshold of tympanometry. However, middle ear function is not specifically considered in this definition. The present study aimed to prospectively collect audiometric data for normal hearing that are based not only on tone audiometry but also supplemented by objective data on middle ear function.

**Methods:**

The examinations were carried out in 84 ears of test subjects aged 18–25 years with normal hearing according to tone audiometry. Objective middle ear diagnostics were performed using classic tympanometry, broadband absorbance tympanometry, and stapedius reflex threshold measurement. A distortion product otoacoustic emission (DPOAE) measurement was also carried out.

**Results:**

Compliance of more than 2 mmho was found in 7 of the 84 ears examined, which are considered separately in the following. The resonance frequency of these 7 ears was below the first quartile of the comparison group (759 Hz), and the absorbance of this group was above the third quartile of the absorbance of the group with regular compliance for frequencies up to 750 Hz. In the group with increased compliance, in more cases than in the comparison group (14% vs. 5%) were no DPOAEs detectable at at least one frequency. In 6 of the 7 ears with increased compliance, stapedius reflex thresholds were found within the whisker range of the group with regular compliance at all measured frequencies.

**Conclusion:**

In the DIN standard for classification of normal hearing, the definition of normal hearing is based solely on tone audiometric parameterization. The reference values measured in this study with regard to compliance of the eardrum show clear inter-individual variance. Supplementing the assessment of middle ear function, such as by measuring compliance, resonance frequency, and/or absorbance curves, could provide additional information to confirm normal hearing.

In der HNO-Heilkunde sind Untersuchungen an normalhörenden Personen zur Gewinnung von Norm- oder Referenzwerten immer wieder von Interesse. Dies kann die Einführung neuer Therapieverfahren oder den Einsatz innovativer Diagnostikmethoden betreffen. Um pathologische Befunde sicher erkennen und einordnen zu können, ist hierfür die Kenntnis des „Normalen“ unabdingbar. Im klinischen Sinne kann eine Normalhörigkeit durch den kombinierten Einsatz von subjektiven und objektiven Messverfahren abgesichert werden. Hierzu zählen Ton- und Sprachaudiometrie, Tympanometrie und Stapediusreflexmessung sowie die Messung von otoakustischen Emissionen und akustisch evozierten Potenzialen [1].

Bei wissenschaftlichen Fragestellungen basiert die Definition der Normalhörigkeit hingegen überwiegend auf dem etablierten Ansatz, wie er in der internationalen Norm für Sprachaudiometrie aufgeführt ist. Die Definition der Normalhörigkeit bezieht sich hier ausschließlich auf die Luftleitungsschwelle der Tonaudiometrie [2]. Die Betrachtung der Mittelohrfunktion mithilfe von objektiven audiometrischen Untersuchungen wird bei diesem Vorgehen nicht berücksichtigt. Im klinischen Alltag wird jedoch mitunter beobachtet, dass Personen mit einem normalen tonaudiometrischen Befund auch untypische Befunde in der Tympanometrie aufweisen können, beispielsweise abgeflachte Kurvenverläufe, welche mit einer Tympanosklerose assoziiert sein können [3].

Bei der klassischen Tympanometrie wird der Kehrwert des Widerstands am Trommelfell, die Admittanz, gemessen, indem ein Sondenton in den äußeren Gehörgang abgegeben wird [4]. Die Admittanz kann als Leichtigkeit verstanden werden, mit der der Trommelfell-Mittelohr-Apparat den Schall weiterleitet [5]. Bei Messung mit der standardmäßigen Messfrequenz von 226 Hz entspricht die gemessene Admittanz, die Ohren von neugeborenen Kindern ausgenommen, der Compliance (Nachgiebigkeit) an der Sondenspitze im äußeren Gehörgang. Dieser Wert kann nicht unmittelbar zur Diagnostik des Mittelohrs herangezogen werden, da er die kombinierte Compliance des Mittelohrs und des Luftvolumens zwischen Messsonde und dem Trommelfell darstellt. Zwar wird der Einfluss des Volumens im Gehörgang messtechnisch durch Über- oder Unterdruck kompensiert, allerdings wird damit verständlich, dass die Aussagekraft der gemessenen Compliance wesentlich von der Platzierungstiefe der Messsonde im Gehörgang abhängt. Die klassische Tympanometrie ist weiterhin darin limitiert, dass sie keine spezifischen Informationen über die jeweiligen Strukturen und Pathologien des Mittelohrs liefert und ihre Aussagekraft auf die jeweilige einzelne Messfrequenz beschränkt ist [6]. Zudem ist der Bereich der Messfrequenz, und damit auch die frequenzbezogene Aussagekraft der Tympanometrie, aufgrund von stehenden Wellen im Gehörgang begrenzt [7].

Als methodische Alternative zur klassischen Tympanometrie steht die Breitband-Tympanometrie zur Verfügung. Hierbei wird das Reflexionsvermögen am Trommelfell als Antwort auf einen Klick-Stimulus in einem breiten Frequenzbereich von 226 bis 8000 Hz bei gleichzeitiger Druckänderung im äußeren Gehörgang gemessen. Die Messergebnisse können entweder als Reflexionsvermögen oder als Absorbanz (Absorptionsvermögen des Mittelohrs von Schall) dargestellt werden, wobei beide Größen Werte zwischen 0 und 1 annehmen können. Am Beispiel der Absorbanz wird bei einem Wert von 1 davon ausgegangen, dass der Schall vollständig vom Mittelohr absorbiert wurde, während ein Wert von 0 darauf hindeuten kann, dass der Schall vollständig am Trommelfell reflektiert worden ist.

Im Gegensatz zur klassischen Tympanometrie lassen sich die methodischen Einschränkungen durch die Platzierungstiefe der Messsonde bei der Breitband-Tympanometrie besser kontrollieren, wodurch genauere Ergebnisse erzielt werden können [7]. Zudem werden durch den erweiterten Frequenzbereich zusätzliche Daten zur Mittelohrfunktion bei vergleichbarer Messdauer generiert. Bereits in vorherigen Studien konnte ein klinischer Nutzen der breitbandigen Absorbanzmessung gezeigt werden, um Mittelohrpathologien zu identifizieren [7–10].

Die Motivation für diese Arbeit war es, die unter Verwendung der klassischen und der Breitband-Tympanometrie gewonnenen Daten zum Status des Mittelohrs zu nutzen, um einen normalen Mittelohrbefund zu definieren. Wir stellten die Hypothese auf, dass die alleinige Definition der Normalhörigkeit über tonaudiometrische Kenndaten nicht ausreicht, um eine Normalhörigkeit umfassend charakterisieren zu können. Dies sollte durch die Ergänzung der Tonaudiometrie um die klassische Tympanometrie, die breitbandige Absorbanzmessung sowie Stapediusreflexschwellen- und Messungen der distorsiv produzierten otoakustischen Emissionen (DPOAE) an einem normalhörenden Kollektiv nach DIN 8253:3 untersucht werden.

## Methode

### Probandinnen und Probanden

Die vorliegende Studie wurde mit Probandinnen und Probanden, welche anamnestisch, ohrmikroskopisch und tonaudiometrisch als normalhörend eingestuft worden sind, durchgeführt. Die Erhebung der Anamnese erfolgte mithilfe eines strukturieren Fragebogens vor der klinischen Untersuchung. Um die anamnestischen Kriterien der Normalhörigkeit zu erfüllen, mussten die Antworten auf Fragen in Bezug auf vorherige Erkrankungen und Voroperationen des Ohrs unauffällig sein. Des Weiteren erfolgte am Tag der Messung eine ärztliche otoskopische Untersuchung, in der ausschließlich unauffällige Normalbefunde in die Studie einbezogen wurden. Sämtliche pathologischen Veränderungen des Trommelfells oder des äußeren Gehörgangs führten zum Ausschluss. Für die Tonaudiometrie wurde das Kriterium für Normalhörigkeit aus der DIN-EN-ISO-8253:3-Norm [2] herangezogen. Normalhörende erfüllen dieses Kriterium mit einer Luftleitungsschwelle bei ≤ 10 dB HL an den Oktavfrequenzen zwischen 125 und 8000 Hz, zudem darf der Hörverlust bei zwei Frequenzen nicht größer als 15 dB HL sein. Die Studienteilnehmenden wurden sowohl schriftlich als auch in einem persönlichen Gespräch vor der Teilnahme zur Studie aufgeklärt. Für die Studie lag ein positives Ethikvotum vor (D 410/23).

Es wurden 49 Personen zwischen 18 und 25 Jahren für eine Teilnahme an der Studie rekrutiert. Von 7 Personen dieser Gruppe wurden die Kriterien der Normalhörigkeit entsprechend der DIN-Norm 8253:3 nicht erfüllt. Letztendlich konnten 42 Personen mit 84 Ohren in die Studie eingeschlossen werden (Altersmittelwert 23,3 Jahre, ± Standardabweichung 1,5 Jahre; 31 weibliche Probandinnen und 11 männliche Probanden).

### Messdurchführung

Für die prospektive Studie kam folgendes Messprotokoll zum Einsatz, wobei alle Messungen an einem Untersuchungstag nacheinander durchgeführt wurden:

1. Erhebung der Anamnese mithilfe eines strukturierten Fragebogens
2. Otoskopie
3. Tonaudiometrie mit einem Exquinox-Audiometer der Fa. Interacoustics A/S (Middelfart, Dänemark). Als Messprogramm diente evidENT 3 (Fa. Merz Medizintechnik, Reutlingen, Deutschland). Die Hörschwelle über Luftleitung wurde für 11 Frequenzen bestimmt (In Hz: 125; 250; 500; 750; 1000; 1500; 2000; 3000; 4000; 6000; 8000) sowie die Knochenleitung an allen Oktavfrequenzen zwischen 250 und 4000 Hz.
4. Klassische Tympanometrie bei 226 Hz, mit kontinuierlicher Druckänderung von 50 daPa/s zwischen 300 und −300 daPa
5. Bestimmung der Stapediusreflexschwelle bei 500, 1000, 2000 und 4000 Hz, gemessen mit einem Startpegel von 60 dB. Der Prüfpegel wurde in 5‑dB-Schritten erhöht bis zum Nachweis des Stapediusreflexes oder bis zum Erreichen der technischen Messgrenze (500 Hz: 100 dB; 1000 Hz: 105 dB; 2000 Hz: 105 dB; 4000 Hz: 100 dB).
6. Messung von distorsiv produzierten otoakustischen Emissionen (DPOAE). Die DPOAE wurden für 1000, 2000, 3000, 4000 und 6000 Hz bestimmt. Die Grenze des Nachweises für eine positive Antwort der DPOAE liegt bei einem DPOAE-Pegel oberhalb −10 dB in Verbindung mit einem SNR von > 6 dB.
  In dieser Studie wurden keine transitorisch evozierten otoakustischen Emissionen, sondern ausschließlich DPOAE gemessen, da DPOAE im Allgemeinen empfindlicher und frequenzspezifischer sind als transitorisch evozierte otoakustische Emissionen [11].
7. Breitband-Absorbanz-Tympanometrie mit Titan-Sonde der Fa. Interacoustics A/S. Die Messung erfolgte mit einer Druckänderungsgeschwindigkeit von 60 daPa/s zwischen 300 und −300 daPa unter Verwendung eines Klick-Stimulus (226 und 8000 Hz).

### Datenanalyse und Statistik

Die generierten Messdaten wurden weiterführend mit Excel (Fa. Microsoft, Redmond, WA, USA) und MATLAB^TM^ (The MathWorks, Inc., Natick, MA, USA) analysiert. Bei der Darstellung der Messergebnisse als Boxplots wurden der Median, das erste und dritte Quartil, die Whisker und der Ausreißer aufgezeigt [12]. Ausreißer wurden als Werte definiert, die den 1,5-fachen Interquartilsabstand (Abstand zwischen dem ersten und dritten Quartil) überschreiten.

**Abb. 1:**
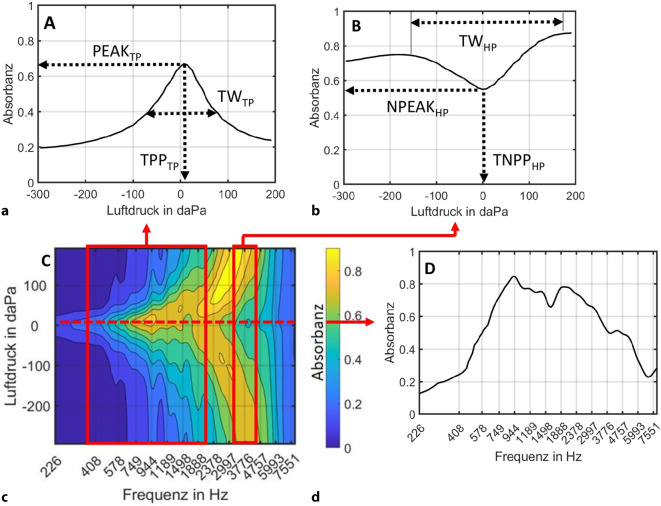
Reduzierung der gemessenen dreidimensionalen Absorbanz (**c**) auf zweidimensionale Verläufe und einzelne Kenndaten. **a** Mittelung der druckabhängigen Absorbanz über den Frequenzbereich von 380 bis 2000 Hz (Tiefpass-Absorbanz-Tympanogramm). **b** Mittelung der druckabhängigen Absorbanz über den Frequenzbereich von 3000 bis 4600 Hz (Hochpass-Absorbanz-Tympanogramm). **d** Frequenzverlauf der Absorbanz zwischen 226 und 8000 Hz bei tympanometrischem Spitzendruck. Die beispielhaften Kurvenverläufe der druckabhängigen Absorbanz sind mit spezifischen Parametern gekennzeichnet

Im Rahmen dieser Studie wurden folgende Variablen analysiert:

1. Klassische Tympanometrie bei 226 Hz:
  - Compliance
  - Tympanometrischer Spitzendruck
  - Gradient
2. Stapediusreflexmessung: *Schwellenwerte* bei 500, 1000, 2000 und 4000 Hz. Waren keine Reflexe bis max. Stimulationspegel messbar, wurde dies mit einem Wert von 130 dB notiert.
3. Messung von DPOAE: *Nachweisbarkeit entsprechend der oben angegebenen Kriterien*
4. Breitband-Absorbanz-Tympanometrie. Im Zuge der Auswertung wurden die dreidimensionalen Messdaten der Absorbanz (Abb. 1c) auf zweidimensionale Daten wie folgt reduziert: die Tiefpass- und Hochpass-Absorbanz-Tympanogramme wurden zur Analyse der Absorbanz in Abhängigkeit vom Luftdruck erstellt. Es wurde die Absorbanz über die gemessenen Frequenzen (Abb. 1a) zwischen 380 und 2000 Hz angelehnt an Liu et al. [13] bzw (Abb. 1b). zwischen 3000 und 4600 Hz gemittelt und in Abhängigkeit vom Druck (−300 bis +200 daPa) dargestellt. Aus diesen bandpassgefilterten Absorbanz-Tympanogrammen wurden weiterführend Kenngrößen extrahiert. Im Tiefpass-Absorbanz-Tympanogramm wurden die maximale Absorbanz *PEAK*_*TP*_, der tympanometrische Spitzendruck bei maximaler Absorbanz (*TPP*_*TP*_) sowie die tympanometrische Breite |*TW*_*TP*_| ermittelt. Letztere ist der Betrag der Druckdifferenz zwischen dem linken und rechten Kurvenabfall bei 75 % der maximalen Absorbanz [14]. Die Betrachtung des Hochpass-Absorbanz-Tympanogramms erfolgte auf der Grundlage des Kurvenverlaufs. Lag eine zweigipflige, „M“-förmige Kurve vor, wurden drei Parameter bestimmt: Das lokale Minimum der Absorbanz NPEAK_HP_ mit dem zugehörigen Druckwert *TNPP*_*HP*_ sowie der tympanometrischen Breite |*TW*_*HP*_|. Die tympanometrische Breite wurde aus dem Abstand der beiden lokalen Maxima berechnet.
  Zur Auswertung der frequenzabhängigen Absorbanz wurde der Frequenzverlauf zwischen 226 und 8000 Hz bei tympanometrischem Spitzendruck betrachtet (Abb. 1d). Zudem wurde die Resonanzfrequenz bestimmt. Aus dem 3‑D-Absorbanztympanogramm lässt sich die Resonanzfrequenz abschätzen, indem die Grenzfrequenz zwischen eingipfligem und mehrgipfligem Druckverlauf der Absorbanz identifiziert wird.

## Ergebnisse

### A-priori-Klassifizierung auf Grundlage der klassischen Tympanometrie

**Abb. 2:**
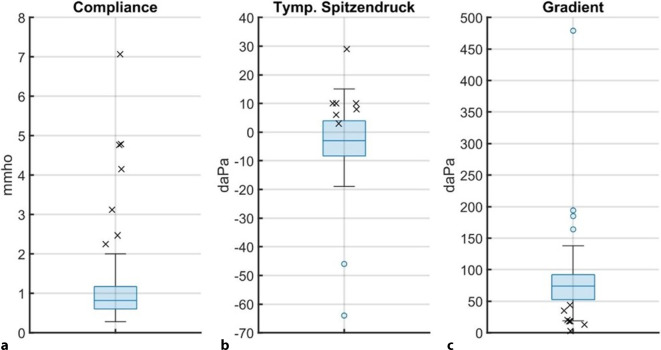
Ergebnisse der klassischen Tympanometrie mit Messfrequenz 226 Hz. Darstellung als *Boxplots* für die *n* = 77 Ohren, die in der Tympanometrie eine Compliance ≤ 2 mmho aufweisen; für *n* = 7 Ohren mit einer Compliance > 2 mmho sind die Werte mit je einem *Kreuz* eingefügt; Ausreißer sind mit einem *Kreis* markiert

Für alle 84 untersuchten Ohren konnten die Variablen Compliance, tympanometrischer Spitzendruck und Gradient aus dem Tympanogramm bestimmt werden (Abb. 2). Im Rahmen der Auswertung erfolgte eine Klassifizierung der Proband*innen anhand der Compliance. Der 90 %-Bereich für Normalwerte der Compliance kann, bezogen auf alle Altersgruppen, zwischen 0,3 und 2 mmho eingeordnet werden [15]. Auf dieser Grundlage wurden die Daten oberhalb einer Compliance von 2 mmho im Weiteren nicht ausgeschlossen, sondern separat betrachtet. Die sieben Ohren mit einer höheren Compliance als 2 mmho (Gruppe „erhöhte Compliance“) wurden in den Grafiken mit einem Kreuz markiert. Bei den 77 Ohren mit einer Compliance ≤ 2 mmho (Gruppe „reguläre Compliance“) war die Compliance im Median bei 0,82 mmho (1. Quartil 0,61; 3. Quartil 1,17).

Der Median des Spitzendrucks beträgt −3 daPa (1. Quartil −8 daPa; 3. Quartil 4 daPa) und der Median des Gradienten 74 daPa (1. Quartil 53 daPa; 3. Quartil 92 daPa), für die 77 Ohren mit einer Compliance ≤ 2 mmho. Bei sechs der sieben Ohren aus der Gruppe mit erhöhten Compliance befindet sich der Spitzendruck-Wert oberhalb des 3. Quartils für die Gruppe mit regulärer Compliance. Alle Gradient-Werte der sieben Ohren mit erhöhter Compliance befinden sich unterhalb des 1. Quartils der Gruppe mit regulärer Compliance.

### Stapediusreflexschwellen

**Abb. 3:**
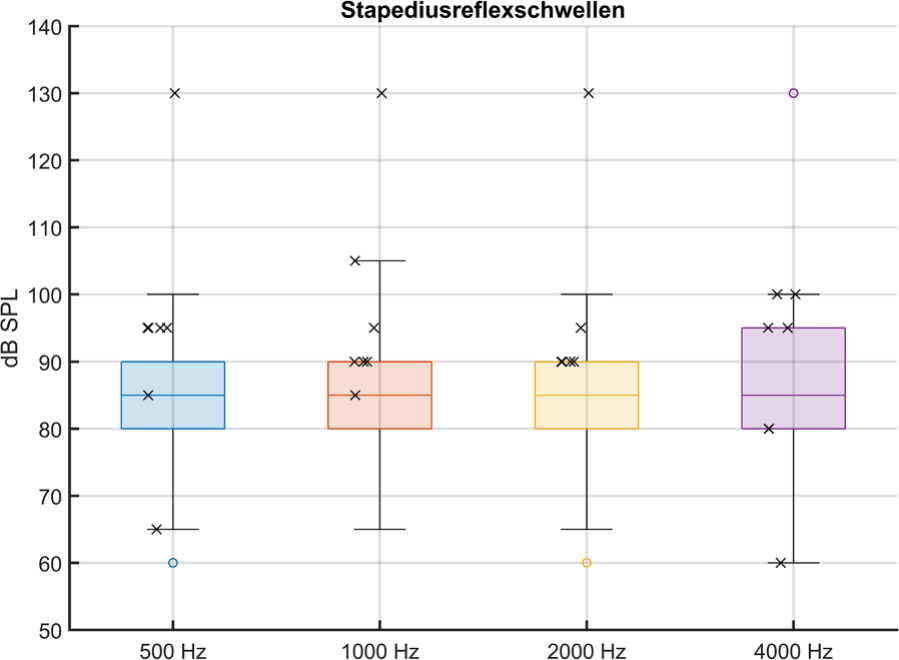
Schwellen der Stapediusreflexbestimmung als *Boxplots* für die *n* = 77 Ohren, die in der Tympanometrie eine Compliance ≤ 2 mmho aufweisen; für *n* = 7 Ohren mit einer Compliance > 2 mmho sind die Werte mit je einem *„X“* eingefügt; Ausreißer sind einem *Kreis* markiert

Die Ergebnisse der Stapediusreflexschwellen-Messung sind in Abb. 3 dargestellt. In der Gruppe mit regulärer Compliance konnten für 500, 1000 und 2000 Hz bei allen 77 Ohren Stapediusreflexe nachgewiesen werden. Für 4000 Hz waren bei 13 der 77 Ohren (16,9 %) keine Stapediusreflexe messbar. Der Median beträgt bei allen vier untersuchten Frequenzen 85 dB SPL. In der Gruppe mit erhöhter Compliance lagen die Stapediusreflexschwellen bei sechs von sieben Ohren für alle vier Messfrequenzen innerhalb des Whisker-Bereichs der Vergleichsgruppe mit regulärer Compliance; bei 4000 Hz traf dies auf alle sieben Ohren zu. Bei einem weiteren Ohr mit erhöhter Compliance fanden sich bei drei Frequenzen (500, 1000, 2000 Hz) keine auslösbaren Stapediusreflexe, jedoch war für 4000 Hz ein Reflex messbar, dessen Schwelle sich im Streubereich der Schwellen für die Gruppe mit regulärer Compliance befand.

### Distorsiv produzierte otoakustische Emissionen (DPOAE)

Alle 77 Ohren mit regulärer Compliance wiesen bei mindestens einer gemessenen Frequenz nachweisbare DPOAE auf. Bei vier Ohren aus dieser Gruppe (5,2 %) waren bei mindestens einer Frequenz keine DPOAE nachweisbar. In der Gruppe mit erhöhter Compliance fanden sich bei einem der sieben Ohren bei mindestens einer Frequenz keine nachweisbaren DPOAE. Dies entspricht einer Häufigkeit von 14,3 % für diese Gruppe.

### Breitband-Absorbanz-Tympanometrie

**Abb. 4:**
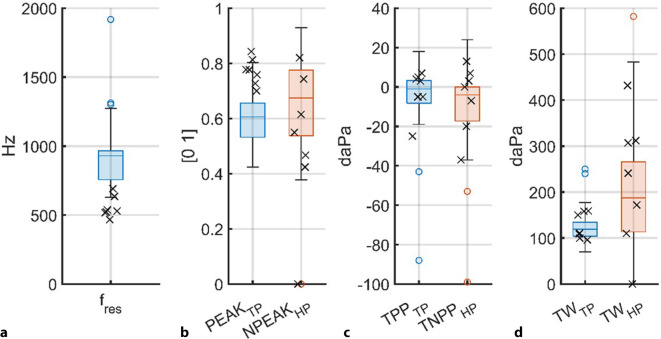
Ergebnisse der Breitband-Absorbanz-Tympanometrie entsprechend der Parametrisierung in Abb. 1. **a** Resonanzfrequenz f_res_. **b** Maximale Absorbanz *PEAK* im Tiefpass-Tympanogramm und lokales Minimum der Absorbanz *NPEAK* im Hochpass-Tympanogramm. **c** Tympanometrischer Spitzendruck *TPP* und *TNPP* im Tief- bzw. Hochpass-Tympanogramm. **d** Tympanometrische Breite *TW* im Tiefpass- bzw. Hochpass-Tympanogramm. Darstellung als *Boxplots* im Tiefpass-Tympanogramm (*TP*) für *n* = 77 Ohren mit eingipfligem Verlauf und einer Compliance ≤ 2 mmho sowie im Hochpass-Tympanogramm (*HP*) für *n* = 67 Ohren mit zweigipfligem Verlauf. Die Messwerte der *n* = 7 Ohren mit erhöhter Compliance (> 2 mmho) im Tiefpass- und der *n* = 6 entsprechenden Ohren im Hochpass-Tympanogramm sind jeweils mit einem *„X“* markiert. Ausreißer sind durch *Kreise* gekennzeichnet

In Abb. 4 sind Parameter der Breitband-Absorbanz-Tympanometrie als Boxplots dargestellt. Die Resonanzfrequenz (Abb. 4a) in der Gruppe mit regulärer Compliance lag im Median bei 930 Hz (1. Quartil: 759 Hz; 3. Quartil: 966 Hz). Die Resonanzfrequenz aller sieben Ohren mit erhöhter Compliance befand sich unterhalb des ersten Quartils der Vergleichsgruppe.

Die Voraussetzung für die Parametrisierung im Hochpass-Tympanogramm ist eine zweigipflige M‑Konfiguration. Für das Hochpass-Tympanogramm erfolgte die Berechnung der folgenden Parameter für die 67 Ohren mit einem zweigipfligen Tympanogramm. Von den 77 Ohren wiesen 10 kein zweigipfliges Tympanogramm auf. Deshalb konnten hier die Parameter NPEAK, TNPP und TW nicht bestimmt werden. Sechs Ohren zeigten ein eingipfliges Hochpass-Tympanogramm, vier Ohren ein dreigipfliges Muster. Von den Ohren mit erhöhter Compliance (> 2 mmho) wiesen 6 der 7 Ohren eine zweigipflige Form im Hochpass-Tympanogramm auf, ein Ohr zeigte eine dreigipflige Form.

Zudem wurden die maximale Absorbanz *PEAK*_*TP*_ des Tiefpass-Tympanogramms und das lokale Minimum der Absorbanz *NPEAK*_*HP*_ des Hochpass-Tympanogramms bestimmt (Abb. 4b). Die *PEAK*_*TP*_-Werte, normiert auf den Wertebereich [0;1], zeigten einen Median von 0,61 (1. Quartil: 0,53; 3. Quartil: 0,66). Hierbei lagen alle Werte der Proband*innen mit erhöhter Compliance oberhalb des dritten Quartils. Im Hochpass-Tympanogramm betrug der Median der *NPEAK*-Werte 0,70 (1. Quartil: 0,61; 3. Quartil: 0,78). Die Werte der Gruppe mit erhöhter Compliance verteilten sich über den gesamten Bereich, ohne erkennbare Häufung innerhalb eines bestimmten Intervalls.

Für den Spitzendruck *TPP*_*TP*_ im Tiefpass-Tympanogramm ergab sich ein Median von −1 daPa, mit einem Wert von −8 daPa fürs 1. Quartil und 3 daPa für 3. Quartil (Abb. 4c). Der *TNPP*_*HP*_ zeigte einen Median von −8 daPa (1. Quartil: −20 daPa; 3. Quartil: 0 daPa). Die Werte der Gruppe mit erhöhter Compliance verteilten sich über den gesamten Bereich beider Gruppen, ohne erkennbare Tendenz zur Häufung in einem bestimmten Abschnitt.

Die *TW*_*TP*_-Werte (Abb. 4d) im Tiefpass-Tympanogramm wiesen einen Median von 119 daPa auf (1. Quartil: 104 daPa; 3. Quartil: 134 daPa). Die *TW*_*HP*_-Werte lagen im Median bei 209 daPa (1. Quartil: 146 daPa; 3. Quartil: 271 daPa). Es zeigte sich eine gleichmäßige Verteilung der Werte der Gruppe mit erhöhter Compliance über den gesamten Wertebereich, ohne Anzeichen einer Schwerpunktbildung.

**Abb. 5:**
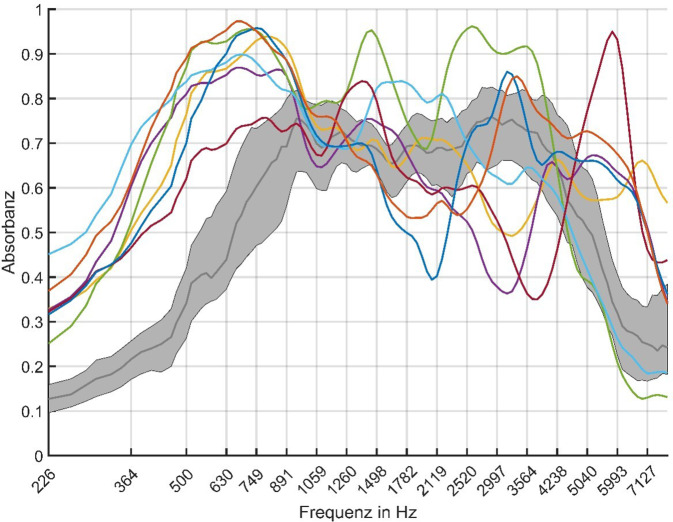
Frequenzverlauf der Absorbanz bei tympanometrischem Spitzendruck. Dargestellt sind der Verlauf für *n* = 77 Ohren mit regulärer Compliance ≤ 2 mmho in der klassischen Tympanometrie (Median in *Dunkelgrau*, *grauschattierter Bereich* zeigt den Bereich von 1.–3. Quartil), sowie *n* = 7 Ohren mit Compliance > 2 mmho (*farbige Einzelkurven*)

In Abb. 5 dargestellt sind der Median (schwarze Kurve) sowie das 1. und 3. Quartil (grauschattierter Bereich) des Frequenzgangs der Absorbanz bei tympanometrischem Spitzendruck für die Gruppe mit regulärer Compliance. Die Frequenzverläufe der sieben Ohren mit erhöhter Compliance sind separat illustriert (farbige Einzelkurven). Bei diesen Ohren befand sich die Absorbanz für Frequenzen bis 750 Hz, entsprechend der überhöhten Compliance bei der klassischen Tympanometrie, über dem 3. Quartil der Absorbanz für die Gruppe mit regulärer Compliance. Für hohe Frequenzen wiesen alle Ohren aus der Gruppe mit überhöhter Compliance Absorbanzwerte auf, die vom Streubereich der Gruppe mit regulärer Compliance abwichen, wobei diese Abweichungen im Frequenzverlauf und in ihrer Ausprägung variierten.

## Diskussion

Ein bewährtes Instrument zur Kategorisierung von Probandengruppen in Studien stellt die Definition der Normalhörigkeit nach DIN-Norm 8253:3 dar. Die Definition der Normalhörigkeit bezieht sich, gemäß dieser internationalen Norm für Sprachaudiometrie [2], ausschließlich auf die Luftleitungsschwelle in der Tonaudiometrie. Rosowski et al. zeigten, dass sich zwischen objektiven Parametern der Mittelohrmechanik und subjektiv ermittelten Luftleitungsschwellen kein signifikanter Zusammenhang feststellen lässt [16]. Dies deutet darauf hin, dass audiometrisch normale Hörschwellen nicht notwendigerweise mit einer normalen Mittelohrfunktion einhergehen. Die objektive Betrachtung der Mittelohrfunktion findet in der DIN-Norm der Normalhörigkeitsdefinition jedoch keine Berücksichtigung. In der vorliegenden Studie wurden zusätzlich zur Luftleitungshörschwelle objektive audiometrische Kenndaten an einem Kollektiv von normalhörenden Proband*innen gewonnen. Es wurde untersucht, ob es bei diesen Daten zu Abweichungen von der Normalhörigkeit innerhalb des Kollektivs gekommen ist.

### Studieneinschluss

Für die Studie wurden Personen im Alter von 18 bis 25 Jahren rekrutiert, die sich selbst als subjektiv normalhörend bezeichneten und eine unauffällige Anamnese in Bezug zum Hören aufwiesen. Im Verlauf des Probandeneinschlusses in die Studie wurde beobachtet, dass es bei der Einschätzung des Hörvermögens und dem tonaudiometrischen Status zu einer entscheidenden Abweichung im Hinblick auf Normalhörigkeit kommen kann. Sieben Personen, welche anamnestisch und otoskopisch als unauffällig klassifiziert wurden, erfüllten jedoch nicht die tonaudiometrischen Voraussetzungen nach internationaler Norm für Sprachaudiometrie [2], um als normalhörende Personen in diese Studie eingeschlossen werden zu können. Eine vergleichbare Diskrepanz zwischen subjektiver und objektiver Hörwahrnehmung wurde bereits in früheren Studien dokumentiert [17].

### Klassifizierung der Mittelohrfunktion mithilfe objektiver Daten

Bei der Analyse der Messdaten der Studienteilnehmer*innen wurde bei sieben Ohren trotz normaler tonaudiometrischer Werte eine Compliance festgestellt, die oberhalb des 90. Perzentils der in der Literatur beschriebenen Normwerte (0,3 bis 2 mmho) lag [15]. Da die Compliance ein etabliertes Maß zur Charakterisierung eines klassischen Tympanogramms bei 226 Hz darstellt, wurde es hier dazu verwendet, das untersuchte Kollektiv an Proband*innen in solche mit regulärer Compliance (≤ 2 mmho) und erhöhter Compliance (> 2 mmho) aufzuteilen, weshalb die weitere Analyse der Messdaten für diese beiden Gruppen getrennt erfolgte.

Dies wirft die Frage auf, ob die sieben der 84 Ohren (8,3 %) mit auffällig hoher Compliance tatsächlich dem oberen Bereich der als normal eingestuften Verteilung – also oberhalb des 90. Perzentils – zuzuordnen sind oder ob der bestehende Referenzbereich gegebenenfalls überdacht bzw. erweitert werden sollte. Eine erhöhte Compliance im Tympanogramm kann auf eine übermäßige Beweglichkeit der Gehörknöchelchenkette oder des Trommelfells hinweisen [18]. Damit könnte bei einer erhöhten Compliance an mögliche subtile Pathologien gedacht werden, z. B. eine Luxation oder Dislokation der Ossikel, sowie eine atrophische Veränderung des Trommelfells durch Ausdünnung oder Vernarbung. Für eine pathologische Ursache der erhöhten Compliance spricht, dass die betroffenen sieben Ohren auch in weiteren Messungen auffällige Werte aufwiesen. Es wurde beobachtet, dass bei Ohren mit einer Compliance über 2 mmho häufiger für mindestens eine Frequenz keine DPOAE nachgewiesen werden konnte (14,3 %) als bei Ohren mit einer Compliance kleiner oder gleich 2 mmho (5,2 %). Dies kann damit erklärt werden, dass Variationen im äußeren und mittleren Ohr die Übertragungseigenschaften des Mittelohrs verändern und damit die Nachweisbarkeit der otoakustischen Emissionen beeinflussen [19]. Otoakustische Emissionen können jedoch auch bei Patient*innen mit unauffälliger Mittelohrfunktion ausbleiben. Daher können diese Tests zu falsch-positiven Ergebnissen führen. Insbesondere DPOAE können durch eine Funktionsstörung der Eustachischen Röhre beeinflusst werden, die zu positivem oder negativem Druck führt [9]. Dennoch zeigten 6 von 7 Ohren nachweisbare Stapediusreflexe und DPOAE, lediglich ein Ohr ist in beiden Messverfahren auffällig.

### Erweiterte Mittelohrdiagnostik durch breitbandige Absorbanzmessung

Bei der Unterscheidung der Mittelohrfunktion anhand der tympanometrischen Compliance sollte bedacht werden, dass eine überhöhte Compliance auch aus einer Fehlmessung durch z. B. inadäquate Sondenplatzierung im Gehörgang resultieren kann. Diese Erklärung scheint für die sieben Ohren mit überhöhter Compliance nicht nachvollziehbar, da sich bei diesen Fällen auch in der Absorbanzmessung ein klarer Trend zeigte: Die Resonanzfrequenzen sämtlicher sieben Ohren mit erhöhter Compliance lagen unterhalb des ersten Quartils der Vergleichsgruppe (759 Hz) mit regulärer Compliance und wiesen bis zu einer Frequenz von 750 Hz eine erhöhte Absorbanz auf (im Frequenzbereich von 257 bis 578 Hz oberhalb der 95. Perzentile der Vergleichsgruppe). Bei Mittelohrerkrankungen, die die Steifheit des Mittelohrs verringern, wie etwa Gehörknöchelchen-Diskontinuität, ist die Absorbanz bei niedrigen Frequenzen erhöht, bei Mittelohrerkrankungen, die die Steifheit des Mittelohrs erhöhen, wie etwa Otosklerose, ist die Absorbanz bei niedrigen Frequenzen reduziert [20]. Veränderte Absorbanzwerte sowie Veränderungen der Resonanzfrequenz können demnach auf veränderte mechanische Eigenschaften des Mittelohrs hindeuten. Der diagnostische Nutzen der breitbandigen Tympanometrie zur Mittelohrdiagnostik und Differenzialdiagnostik von Mittelohrpathologien konnte bereits in mehreren Arbeiten nachgewiesen werden [6, 9, 10, 20–25].

Im Hochtonbereich ab 1000 Hz hingegen zeigten sich deutliche interindividuelle Streuungen der Absorbanz ohne erkennbare systematische Tendenzen. Die bekannte Streuung im Hochfrequenzbereich lässt sich durch die höhere Sensitivität der Messung gegenüber minimalen Veränderungen im Schallleitungssystem erklären [27].

In der Studie von Ayala et al. wurde bei 49 normalhörenden Ohren ein mittlerer tympanometrischer Spitzendruck von −19 daPa (Standardabweichung: 27 daPa) festgestellt. In unserer Untersuchung hingegen lag der Median des TPP bei −3 daPa. Die gemessene Compliance betrug in der Studie von Aithal et al. und dieser Studie jeweils 0,82 mmho. Zudem zeigten alle untersuchten Ohren in beiden Studien bei einer Testfrequenz von 226 Hz ein Tympanogramm vom Typ A, klassifiziert nach Jerger [28]. Diese Übereinstimmungen in den tympanometrischen Parametern unterstreichen die Konsistenz der Messungen und unterstützen die Validität unserer Ergebnisse im Vergleich zu den etablierten Normwerten der Studie von Aithal et al. [24].

Wie auch in der Arbeit von Hougaard et al. gezeigt, hat die druckabhängige Absorbanz im Tieftonbereich, hier zwischen 256 Hz bis 1–2 kHz, einen eingipfligen Verlauf. Bei 2 kHz bis 8 kHz ist kein eindeutiger Peak mehr zu erkennen. Das Tympanogramm beginnt, sich in einem flachen, plateauähnlichen Verlauf auszubreiten [29]. Diese Beobachtungen der Frequenzverläufe der Absorbanz konnten wir auch machen. In der Studie von Hougaard et al. betrug die Resonanzfrequenz 911 Hz (90 %-Bereich: 606,85 bis 1285 Hz). In dieser Arbeit beträgt die Resonanzfrequenz 930 Hz (90 %-Bereich: 674,2 bis 1073,2). Damit zeigen sich in dem Tieftonbereich der Absorbanz und der Resonanzfrequenz nur leichte Abweichungen.

### Auswirkungen

Abschließend stellt sich die Frage, ob die Definition des normalen Hörvermögens künftig unter Einbeziehung der Mittelohrfunktion abgesichert werden sollte. Darüber hinaus bleibt unklar, ob es sich bei den abweichenden Mittelohrfunktionswerten um eine natürliche Streuung innerhalb des statistischen Referenzbereichs handelt oder ob diese bereits auf subklinische Pathologien hinweisen. Die Ergebnisse von Rosowski et al. zeigen, dass die mechanoakustische Nachgiebigkeit des Mittelohrs die Ergebnisse von Absorbanzmessungen bei Frequenzen unter 1 kHz deutlich beeinflussen können, auch wenn die Hörschwellen im Tonaudiogramm unauffällig sind [16].

Damit kann die Hypothese aufgestellt werden, dass die reine Schwellenbestimmung mittels Tonaudiometrie die komplexe Hörphysiologie nicht vollständig abbildet und gegebenenfalls subklinische Mittelohrveränderungen zu veränderten Parametern führen, ohne dabei die Hörschwelle zu beeinträchtigen. Die Ergebnisse dieser Studie unterstreichen die Notwendigkeit einer multimodalen Diagnostik, um subtile oder subklinische Veränderungen im Mittelohr sicher zu erfassen. Die Kombination aus Tympanometrie, DPOAE-Messung und breitbandiger Absorbanzanalyse kann damit eine differenzierte Beurteilung der Schallübertragungskette sowie der cochleären Funktion ermöglichen.

Eine präzise Charakterisierung des normalhörenden Kollektivs würde die Validität von Vergleichsstudien erhöhen und das Risiko von Fehlklassifikationen reduzieren [30]. Ein möglicher Ansatz für zukünftige Studien könnte daher in einer absorbanzgestützten Erweiterung der Kenndaten zur Normalhörigkeit liegen, um genau diese Fragestellungen weiterführend evaluieren zu können.

Auf Grundlage der in dieser Studie erhobenen Daten lassen sich Vorschläge für Referenzwerte der Breitband-Absorbanz-Tympanometrie bei normalhörenden Personen ableiten:

Der typische Verlauf der Absorbanz bei normalhörenden Personen zeigt im Tieftonbereich (etwa 256 Hz bis 1–2 kHz) einen eingipfligen Charakter, während im Hochfrequenzbereich ab etwa 2 kHz eine interindividuelle Streuung der Absorbanzwerte beobachtet wird. Die Resonanzfrequenz liegt dabei bei normaler Mittelohrfunktion in der Regel im Bereich zwischen 674 und 1073 Hz, was dem 90 %-Bereich der gemessenen Werte in dieser Untersuchung entspricht.

## Ausblick

Die in dieser Studie gemessenen Referenzwerte in Bezug auf die Compliance des Trommelfells in der tympanometrischen Messung zeigen eine deutliche interindividuelle Varianz. Mit dieser Beobachtung kann die vorliegende Arbeit zur weiteren klinischen Einschätzung von einem definitionsgemäß normalhörenden Befund beitragen. In der DIN-Norm zur Klassifizierung von Normalhörigkeit besteht die Definition der Normalhörigkeit allein aufgrund tonaudiometrischer Parametrisierung junger Erwachsener [2]. Die Norm 7029 bezieht sich zusätzlich noch auf alters- und geschlechtsspezifische Unterschiede des Hörvermögens [31]. Eine Ergänzung zur Einschätzung des Mittelohrstatus, wie die Messung der Compliance, der Resonanzfrequenz und/oder der Absorbanz-Verläufe, könnte zusätzliche Informationen zur Absicherung einer Normalhörigkeit liefern.

Diese Untersuchung kann somit einen Beitrag liefern für die Diskussion des Einbezugs der ergänzenden Mittelohrdiagnostik wie z. B. der breitbandigen Absorbanzmessung bei der Untersuchung mit Normalhörenden.

## Fazit für die Praxis

- Die alleinige Bestimmung der Normalhörigkeit basierend auf tonaudiometrischen Messdaten der Luftleitung bedingt nicht immer eine normale Mittelohrfunktion.
- Die breitbandige Absorbanzmessung und die klassische Tympanometrie liefern Kennwerte zur weiterführenden Klassifizierung von Normalhörigkeit, die eine Beurteilung der Mittelohrfunktion beinhalten.
- Besonders die Resonanzfrequenz und breitbandige Absorbanzwerte können die Bestimmung der Compliance mittels klassischer Tympanometrie bei 226 Hz ergänzen.

## Data Availability

Daten können nicht zur Verfügung gestellt werden, da sie im Rahmen des Projekts durch den Sponsor verarbeitet werden.

## References

[1] Lehnhardt E, Laszig R (2009) Praxis der Audiometrie. Thieme, Stuttgart 10.1055/b-002-44901

[2] Deutsches Institut für Normung (2022) 8253–3: Akustik-Audiometrische Prüfverfahren-Teil 3: Sprachaudiometrie. Beuth, Berlin

[3] Forli F, Capobianco S, De Vito A, Bruschini L, Lazzerini F (2025) Issues in the audiological assessment of otosclerosis. Acta Otorhinolaryngol Ital 45(3 Suppl 1):S40 40534441 10.14639/0392-100X-supp1_3-45-2025-A1337 PMC12180548

[4] Mrowinski D, Scholz G, Steffens T (2022) Audiometrie. Thieme, Stuttgart 10.1055/b000000821

[5] Acoustical Society of America (ASA) (2020) American National Standard—Specifications for Instruments to Measure Aural Acoustic Impedance and Admittance (Aural Acoustic Immittance). ANSI/ASA S3.39–1987 (R2020). American Institute of Physics, New York

[6] Şahin MI, Özyürek DD, Vural A, Zararsız G, Ketenci I, Ünlü Y (2022) Can Wideband tympanometry predict the prognosis of Otitis media with effusion? J Audiol Otol. 10.7874/JAO.2021.00633 35538866 10.7874/jao.2021.00633 PMC12046198

[7] Margolis RH, Saly GL, Keefe DH (1999) Wideband reflectance tympanometry in normal adults. J Acoust Soc Am 106(1):265–280 10420621 10.1121/1.427055

[8] Karuppannan A, Barman A (2021) Wideband absorbance tympanometry: a novel method in identifying otosclerosis. Eur Arch Otorhinolaryngol 278(11):4305–4314. 10.1007/S00405-020-06571-X 33388979 10.1007/s00405-020-06571-x

[9] Keefe DH, Archer KL, Schmid KK, Fitzpatrick DF, Feeney MP, Hunter LL (2017) Identifying otosclerosis with aural acoustical tests of absorbance, group delay, acoustic reflex threshold, and otoacoustic emissions. J Am Acad Audiol 28(9):838–860. 10.3766/jaaa.16172 28972472 10.3766/jaaa.16172 PMC5987224

[10] Meng X, Zhu K, Yue J, Han C (2022) The role of wideband tympanometry in the diagnosis of Meniere’s disease. Front Neurol. 10.3389/fneur.2022.808921 35153998 10.3389/fneur.2022.808921 PMC8829324

[11] Stamate MC, Todor N, Cosgarea M (2015) Comparative multivariate analyses of transient otoacoustic emissions and distorsion products in normal and impaired hearing. Clujul Med 88(4):500 26733749 10.15386/cjmed-467 PMC4689244

[12] Dawson R (2011) How significant is a boxplot outlier? J Stat Educ 19(2)

[13] Liu Y‑W, Sanford CA, Ellison JC, Fitzpatrick DF, Gorga MP, Keefe DH (2008) Wideband absorbance tympanometry using pressure sweeps: System development and results on adults with normal hearing. J Acoust Soc Am 124(6):3708–3719 19206798 10.1121/1.3001712 PMC2737248

[14] Mewes A et al (2015) Breitbandige Energie-Absorbanz zur Mittelohrdiagnostik. Z Audiol 54(4):138–147

[15] Steffens T (2020) Verbesserung der Genauigkeit durch quantitative Referenzwerte. HNO Nachr 50(4):22–27

[16] Rosowski JJ et al (2012) Ear-canal reflectance, umbo velocity, and tympanometry in normal-hearing adults. Ear Hear 33(1):19–34 21857517 10.1097/AUD.0b013e31822ccb76 PMC3223546

[17] Berufsgenossenschaft Holz und Metall (2013) Studie zur Gehörentwicklung von lärmexponierten Beschäftigten mit Otoplastiken. BGHM, Mainz (https://www.bghm.de/fileadmin/user_upload/Arbeitsschuetzer/Fachthemen/Laerm_Vibration/Studie_Otoplastiken.pdf)

[18] Grevers G, Iro H, Probst R (2008) Hals-Nasen-Ohren-Heilkunde. Thieme

[19] do Couto CM, Carvallo RMM (2009) The effect external and middle ears have in otoacoustic emissions. Braz J Otorhinolaryngol 75(1):15–23 19488555 10.1016/S1808-8694(15)30826-0 PMC9442167

[20] Shahnaz N, Bork K (2006) Wideband reflectance norms for Caucasian and Chinese young adults. Ear Hear 27(6):774–788 17086086 10.1097/01.aud.0000240568.00816.4a

[21] Kim SY et al (2019) Differentiating among conductive hearing loss conditions with wideband tympanometry. Auris Nasus Larynx 46(1):43–49 29885747 10.1016/j.anl.2018.05.013

[22] Ellison JC, Gorga M, Cohn E, Fitzpatrick D, Sanford CA, Keefe DH (2012) Wideband acoustic transfer functions predict middle-ear effusion. Laryngoscope 122(4):887–894. 10.1002/lary.23182 22374909 10.1002/lary.23182 PMC3432925

[23] Karuppannan A, Barman A (2021) Wideband absorbance pattern in adults with otosclerosis and ossicular chain discontinuity. Auris Nasus Larynx 48(4):583–589. 10.1016/j.anl.2020.10.019 33187789 10.1016/j.anl.2020.10.019

[24] Aithal S, Aithal V, Kei J, Anderson S (2020) Wideband absorbance in ears with retraction pockets and cholesteatomas: a preliminary study. J Am Acad Audiol 31(10):708–718. 10.1055/S-0040-1719130 33588510 10.1055/s-0040-1719130

[25] Feeney MP et al (2017) Normative wideband reflectance, equivalent admittance at the tympanic membrane, and acoustic stapedius reflex threshold in adults. Ear Hear 38(3):e142 28045835 10.1097/AUD.0000000000000399 PMC5404939

[26] Shahnaz N, AlMakadma H, Sanford CA (2023) Assessment of middle ear function using Wideband acoustic Immittance: current practices and future prospects: the rise and fall of aural acoustic Immittance assessment tools. In: Seminars in hearing. Thieme, S 5 10.1055/s-0043-1763298 PMC10014197 36925654

[27] Nakajima HH, Dong W, Olson ES, Merchant SN, Ravicz ME, Rosowski JJ (2009) Differential intracochlear sound pressure measurements in normal human temporal bones. J Assoc Res Otolaryngol 10:23–36 19067078 10.1007/s10162-008-0150-y PMC2644388

[28] Jerger J (1970) Clinical experience with impedance audiometry. Arch Otolaryngol 92(4):311–324 5455571 10.1001/archotol.1970.04310040005002

[29] Hougaard DD, Lyhne NM, Skals RK, Kristensen M (2020) Study on wideband tympanometry and absorbance within a Danish cohort of normal hearing adults. Eur Arch Otorhinolaryngol 277:1899–1905 32172386 10.1007/s00405-020-05909-9

[30] Gorga MP, Neely ST, Ohlrich B, Hoover B, Redner J, Peters JO (1997) From laboratory to clinic: a large scale study of distortion product otoacoustic emissions in ears with normal hearing and ears with hearing loss. Ear Hear 18(6):440–455 9416447 10.1097/00003446-199712000-00003

[31] Michel O (2021) DIN EN ISO 7029: 2017–06: The current DIN thresholds for evaluating normal hearing. HNO 69:1014–1018 34651214 10.1007/s00106-021-01111-3

